# Coronary arterial calcification in rheumatoid arthritis: comparison with the Multi-Ethnic Study of Atherosclerosis

**DOI:** 10.1186/ar2641

**Published:** 2009-03-10

**Authors:** Jon T Giles, Moyses Szklo, Wendy Post, Michelle Petri, Roger S Blumenthal, Gordon Lam, Allan C Gelber, Robert Detrano, William W Scott, Richard A Kronmal, Joan M Bathon

**Affiliations:** 1 Department of Medicine, The Johns Hopkins University, The Johns Hopkins Hospital, 600 N Wolfe Street, Baltimore, MD 21287, USA; 2 Department of Epidemiology, The Johns Hopkins University, The Johns Hopkins Hospital, 600 N Wolfe Street, Baltimore, MD 21287, USA; 3 Department of Radiological Sciences, University of California at Irvine, Medical Sciences Building, Irvine, CA 92697, USA; 4 Department of Radiology, The Johns Hopkins University, The Johns Hopkins Hospital, 600 N Wolfe Street, Baltimore, MD 21287, USA; 5 Department of Biostatistics, University of Washington, F-600, Health Sciences Building, 1705 NE Pacific Street, Seattle, WA 98195, USA; 6 Division of Rheumatology, The Johns Hopkins University, 5200 Eastern Avenue, Suite 4100, Baltimore, MD 21224, USA

## Abstract

**Introduction:**

Although cardiovascular morbidity and mortality are increased in rheumatoid arthritis, little is known about the burden of subclinical coronary atherosclerosis in these patients.

**Methods:**

Using computed tomography, coronary artery calcification was measured in 195 men and women with rheumatoid arthritis aged 45 to 84 years without clinical cardiovascular disease and compared with 1,073 controls without rheumatoid arthritis enrolled in the Baltimore cohort of the Multi-Ethnic Study of Atherosclerosis.

**Results:**

The prevalence of coronary calcification (Agatston score > 0) was significantly higher in men, but not women, with rheumatoid arthritis after adjusting for sociodemographic and cardiovascular risk factors (prevalence ratio = 1.19; *P *= 0.012). Among participants with prevalent calcification, those with rheumatoid arthritis had adjusted mean Agatston scores 53 units higher than controls (*P *= 0.002); a difference greater for men than women (*P *for interaction = 0.017). In all analyses, serum IL-6 attenuated the association between rheumatoid arthritis and coronary calcification, suggesting its role as a potential mediator of enhanced atherosclerosis. Notably, increasing severity of rheumatoid arthritis was associated with a higher prevalence and extent of coronary calcification among both men and women with rheumatoid arthritis, and for all age categories. The largest percentage difference in coronary arterial calcification between rheumatoid arthritis patients and their nonrheumatoid arthritis counterparts was observed in the youngest age category.

**Conclusions:**

Increasing rheumatoid arthritis disease severity was associated with a higher prevalence and greater extent of coronary artery calcification, potentially mediated through an atherogenic effect of chronic systemic inflammation. Gender and age differences in association with coronary calcification suggest that preventive measures should be emphasized in men with rheumatoid arthritis, and considered even in younger rheumatoid arthritis patients with low levels of traditional cardiovascular risk factors.

## Introduction

Cardiovascular disease (CVD) is the leading cause of premature mortality in rheumatoid arthritis (RA) patients [1,2]. Although several studies have suggested that atherosclerosis is increased in RA [3,4], few studies have investigated patients' coronary arteries [5-7]. Coronary arterial calcification (CAC), a subclinical measure of atherosclerosis measured by computed tomography (CT), is associated with the degree of atherosclerotic plaque [8], and is strongly predictive of cardiovascular (CV) events, including those at low risk [9,10]. This is relevant to RA, as most studies have not shown differences in traditional CV risk factors between RA patients and controls [11,12].

Chronic systemic inflammation has been implicated in atherogenesis, and may play a role in destabilizing vulnerable coronary plaques, thereby precipitating acute thrombosis and clinical CV events [13]. Interestingly, atherosclerotic plaque and rheumatoid synovium share an array of inflammatory cells and cytokines [14], suggesting that chronic rheumatoid inflammation may contribute to excess atherosclerosis in RA. In addition, RA may modify the atherogenic effects of noninflammatory risk factors [15], which could explain the higher atherosclerosis risk of RA patients even when their risk factor distributions are the same as those of normal controls. In particular, increasing age and male gender are two of the strongest predictors of CAC [16], yet no previous studies have explored their interactions with RA with regard to CAC.

We designed a comparative cohort study of subclinical atherosclerosis in RA using the Multi-Ethnic Study of Atherosclerosis (MESA) [17] cohort as a control group. This study provided an opportunity to cross-sectionally compare the presence and extent of CAC between RA patients and a subset of the MESA cohort from the same geographic area utilizing the same equipment, laboratories and core reading facilities. We hypothesized that, compared with controls, RA status in general – and increasing RA severity in particular – would be associated with a higher CAC prevalence and extent, and that systemic inflammation would partially account for these associations. We also explored whether differences in CAC between RA and control groups varied by gender, age, and level of RA severity.

## Materials and methods

### Participants and enrollment

#### Rheumatoid arthritis subjects

The Evaluation of Subclinical Cardiovascular disease And Predictors of Events in Rheumatoid Arthritis Study (ESCAPE RA) is a cohort study of the prevalence, progression, and risk factors for subclinical CVD in men and women with RA. The ESCAPE RA study was designed with identical inclusion and exclusion criteria (except for the diagnosis of RA) to MESA, a population-based cohort study of subclinical CVD with similar objectives. The ESCAPE RA inclusion criteria were: fulfillment of American College of Rheumatology criteria for the classification of RA [18] of ≥ 6 months; and age 45 to 84 years. Medical records were reviewed for each participant to confirm diagnosis. Exclusion criteria were: prevalent CVD prior to enrollment (prior CVD was defined as self-reported or physician-diagnosed myocardial infarction, heart failure, coronary artery revascularization, angioplasty, peripheral vascular disease or procedures (excluding varicose vein procedures), implanted pacemaker or defibrillator devices, and current atrial fibrillation); weight exceeding 300 pounds (due to imaging equipment limitations); and CT scan of the chest within 6 months prior to enrollment (to limit radiation exposure).

Given the greater prevalence of RA in women, we set a recruitment goal of at least 40% males to enable gender-specific analyses, and recruited 195 patients from the Johns Hopkins Arthritis Center and by referral from community rheumatologists.

The study was approved by the Johns Hopkins Hospital Institutional Review Board and MESA, with all participants providing informed consent prior to enrollment. Enrollment occurred from October 2004 through May 2006.

#### Control subjects

Non-RA controls were Baltimore MESA study participants. A description of the MESA study design and methods has been published [17]. In brief, MESA enrolled a multi-ethnic cohort of 6,814 participants from six US communities between 2000 and 2002, among whom 1,086 were enrolled by the Johns Hopkins (Baltimore) Field Center. Thirteen controls were excluded for reporting use of disease-modifying antirheumatic drugs (DMARDs) typically used for the treatment of RA.

### Measurement of coronary arterial calcification

All subjects underwent cardiac multidetector row CT scanning using methodology described previously [19]. CAC was quantified using the Agatston method [20], with a phantom of known calcium density scanned along with the participant to ensure standardization across scans [21]. Scans were transmitted electronically to the MESA CT reading center for interpretation. Intra-observer agreement and inter-observer agreement for CT assessors were high (κ = 0.93 and κ = 0.90, respectively).

### Covariate assessment

The ESCAPE RA study used the same questionnaires, equipment, methods, and quality control procedures as MESA. Study coordinators were trained and certified by MESA trainers. Information on demographics, smoking, and family history was collected by questionnaire. Resting blood pressure was measured three times in the seated position, and the average of the last two measurements was used in the analysis. Hypertension was defined by systolic blood pressure ≥ 140 mmHg, diastolic blood pressure ≥ 90 mmHg, or antihypertensive medication use. Diabetes was defined as a fasting serum glucose ≥ 126 mg/dl or use of antidiabetic medications. Physical activity was assessed using the 7-Day Physical Activity Recall questionnaire [22]. The body mass index was calculated as weight (kg) divided by height-squared (m^2^). Waist and hip circumferences were measured with a Gulick II anthropometric measuring tape. Prescription and over-the-counter medications used in the preceding 2 weeks were documented from containers supplied by the participant. Composite CV risk measures were calculated using published criteria for the metabolic syndrome (Adult Treatment Panel III) [23], the Framingham hard 10-year CV risk (National Cholesterol Education Program) [24], and the Reynolds Risk Score (women only) [25].

#### RA-specific covariates

In RA patients, 44 joints were examined by a single trained assessor for swelling, tenderness, deformity, and surgical replacement or fusion. RA disease duration was calculated based on self-report from the time of physician diagnosis. RA activity was calculated using the Disease Activity Score for 28 joints with C-reactive protein (CRP) [26]. Functional limitation was assessed with the Stanford Health Assessment Questionnaire [27]. Current and past use of glucocorticoids and of biologic and nonbiologic DMARDs was ascertained by interview. Single-view, anterior–posterior radiographs of the hands and feet were obtained and scored using the Sharp–van der Heijde method [28] by a single, trained radiologist blinded to patient characteristics. For five subjects with incomplete radiographic assessments, the missing score (hand or foot) was imputed from the available data based on a regression equation using data from the remaining subjects in the cohort.

#### Laboratory covariates

Fasting sera and plasma were separated by centrifugation, and were stored at -70°C. All assays (except RA autoantibodies) were performed at MESA-designated laboratories using MESA quality control procedures. CRP, IL-6, fibrinogen, homocysteine, soluble intracellular adhesion molecule 1 and E-selectin were measured as previously described [29]. Low-density lipoprotein-cholesterol was estimated in plasma specimens having a triglyceride value < 400 mg/dl using the Friedewald equation. Rheumatoid factor was assessed by ELISA, with seropositivity defined at or above a level of 40 units. Anti-cyclic citrullinated peptide antibody was assessed by ELISA, with seropositivity defined at or above a level of 60 units.

### Statistical analysis

All participants with interpretable multidetector row CT scans were included. Means and standard deviations were calculated for normally distributed variables, and medians and interquartile ranges were calculated for non-normally distributed variables. Counts and percentages were calculated for categorical variables. Differences in continuous variables between the RA and control groups were compared using *t *tests (for normally distributed variables) or the Kruskal–Wallis test (for non-normally distributed variables). Categorical variables were compared using the chi-square goodness of fit test or Fisher's exact test. Multivariable linear regression models adjusting for age, gender, and race/ethnicity were constructed to estimate adjusted means, 95% confidence intervals, and *P *values for CV risk factors.

CAC presence was defined as any detectable coronary calcium (Agatston score > 0). Using an alternative cutoff point of 10 Agatston units did not qualitatively change any of the associations observed compared with the 0 unit cutoff point. Multivariate analyses were conducted in participants with complete clinical data (n = 1,222). Poisson regression with robust variance estimation was used to model the association of RA status with the presence of CAC, with prevalence ratios and 95% confidence intervals calculated. Poisson regression was used rather than logistic regression since odds ratios would not approximate prevalence ratios when the outcome is as common as CAC > 0 in the present study (approximately 50%). Risk factors considered included age, gender, race/ethnic background, highest education level attained, systolic and diastolic blood pressures (or presence of hypertension), antihypertensive medication use, high-density lipoprotein and low-density lipoprotein, ever smoking, diabetes, lipid-lowering medication use, fibrinogen, soluble intracellular adhesion molecule, E-selectin, glucose, amount of weekly intentional exercise, and body mass index or waist circumference. Highly skewed variables (for example, triglycerides, fibrinogen) were logarithmically transformed.

Stratified analyses were conducted according to RA severity. We ranked RA patients using propensity scores [30] for current RA disease activity and severity using a model that included the total Sharp–van der Heijde radiographic score, the number of swollen and tender joints, the health assessment questionnaire, minutes of morning stiffness, the cumulative prednisone dose, and the number of past DMARDs prescribed. This method allows each RA subject to be ranked in order from lowest to highest based on the aggregate of their predictor variables. Subjects were then grouped into four ordinal categories: MESA control (no RA severity), and RA low severity, intermediate severity, and high severity based on tertiles of propensity for RA activity and severity. Statistical comparisons of differences in the association of RA status with CAC across subgroups were conducted using the Wald test.

Robust (resistant) regression was used to model the association of RA status with the extent of CAC (as a continuous variable) in patients with CAC > 0, with regression coefficients (β values) and 95% confidence intervals calculated. This method produced estimates similar (to within 1%) to those obtained by linear regression with logarithmically transformed CAC scores and exclusion of extreme outliers (falling above and below two standard deviations of studentized residuals) (data not shown). Robust regression was preferred as it permitted efficiency in the modeling of all data points in subjects with any CAC and yielded meaningful results expressed as absolute means, rather than less meaningful logarithmic means.

Statistical calculations were performed using Intercooled Stata 9 (StataCorp, College Station, TX, USA). In all tests, a two-tailed α value of 0.05 was defined as the level of statistical significance.

## Results

One-hundred and ninety-five RA patients and 1,073 MESA controls underwent multidetector row CT scanning. RA patients tended to have disease of extended duration (median, 9 years), and most (78%) were seropositive for either rheumatoid factor or anti-cyclic citrullinated peptide antibodies and, on average, had evidence of radiographic erosions and deforming arthropathy (Table 1). RA disease activity was low to moderate in most patients. The majority of patients (93%) were treated with DMARDs, including 46% with biologics, either as monotherapy or in combination with a nonbiologic DMARD(s); 40% were currently treated with glucocorticoids, and nearly two-thirds with nonsteroidal anti-inflammatory drugs.

**Table 1:** Baseline disease-related characteristics of rheumatoid arthritis patients: the ESCAPE RA study

Characteristic	Tertiles of propensity for rheumatoid arthritis activity and severity				
	
	All (n = 195)	Tertile 1 (lowest, n = 65)	Tertile 2 (middle, n = 65)	Tertile 3 (highest, n = 65)	*P *value
Disease characteristics					
Disease duration (years)	9 (4 to 17)	4 (2 to 6)	8 (5 to 13)	21 (16 to 31)	<0.001
Rheumatoid factor or anti-cyclic citrullinated peptide seropositivity	152 (78.0)	37 (57.8)	58 (89.2)	53 (86.9)	<0.001
Disease Activity Score (for 28 joints – C-reactive protein)	3.57 (2.87 to 4.35)	3.18 (2.54 to 3.99)	3.57 (2.87 to 4.29)	4.00 (3.35 to 4.88)	<0.001
Health assessment questionnaire score (0 to 3)	0.63 (0.13 to 1.25)	0.13 (0 to 0.50)	0.75 (0.13 to 1.25)	1.38 (1.00 to 1.88)	<0.001
Total Sharp–van der Heijde Score	44 (16 to 116)	18 (5 to 36)	38 (20 to 68)	142 (83 to 221)	<0.001
Deformed + replaced joints	2 (0 to 7)	1 (0 to 2)	1 (0 to 3)	10 (4 to 14)	<0.001
C-reactive protein (mg/l)	2.46 (1.09 to 7.17)	1.7 (0.78 to 4.56)	2.6 (1.18 to 7.80)	2.96 (1.66 to 9.46)	0.004
Current treatment					
Nonbiologic DMARDs only	94 (48.2)	37 (57.8)	29 (44.6)	25 (41.0)	0.19
Biologic DMARD monotherapy	19 (9.7)	5 (7.8)	3 (4.6)	10 (16.4)	
Nonbiologic + biologic DMARDs	70 (35.9)	20 (31.3)	28 (43.1)	22 (36.1)	
No DMARDs	12 (6.2)	2 (3.1)	5 (7.7)	4 (6.6)	
Glucocorticoids	75 (38.5)	23 (35.9)	20 (30.8)	31 (50.8)	0.06
Nonsteroidal anti-inflammatory drugs^a^	126 (64.6)	40 (61.5)	41 (63.1)	45 (69.4)	0.67

Characteristics expressed as median (interquartile range) or *n *(%). DMARD, disease-modifying antirheumatic drug; ESCAPE RA, Evaluation of Subclinical Cardiovascular Disease and Predictors of Events in Rheumatoid Arthritis. ^a^Includes both cyclooxygenase-2 selective and nonselective nonsteroidal anti-inflammatory drugs.

In unadjusted comparisons (Table 2), the RA group was younger and included a higher proportion of women and Caucasians than controls. The RA group also differed significantly from the MESA group in many CV risk factors, including a lower proportion of subjects with diabetes, lower mean fasting glucose, lower prevalence of hypertension despite higher mean diastolic blood pressure, and higher mean high-density lipoprotein-cholesterol concentration. As expected, RA patients had significantly higher median CRP, IL-6, and soluble intracellular adhesion molecule levels than MESA participants. Among composite risk measures, the RA group had a significantly lower mean Framingham 10-year hard CV risk score, lower mean Reynolds Risk Score, and lower proportion of patients who met the Adult Treatment Panel III criteria for metabolic syndrome [23].

**Table 2:** Crude and adjusted baseline characteristics of rheumatoid arthritis patients and controls^a^

	Unadjusted				Adjusted		
	
	RA (n = 195)	MESA (n = 1,073)	*P *value	RA (n = 195)	MESA (n = 1,073)	Adjusted % difference	*P *value
Demographics							
Age (years)	59 ± 9	64 ± 10	<0.001	-	-	-	-
Female	118 (60.5)	526 (49.0)	0.003	-	-	-	-
Race							
Caucasian	169 (85.8)	540 (50.3)	<0.001	-	-	-	-
African American	16 (8.2)	533 (49.7)					
Other	10 (5.1)	0 (0)					
Education							
Some college or higher	147 (75.4)	741 (71.5)	0.26	69.9	73.6	-5.0	0.34
Cardiovascular risk factors							
Diabetes	11 (5.6)	142 (13.2)	0.003	8.1	11.1	-27.0	0.29
Fasting glucose (mg/dl)	93 ± 21	105 ± 31	<0.001	95.9	103.8	-8.2	0.001
Hypertension							
Present^b^	105 (53.6)	597 (55.6)	0.59	64.3	54.3	+18.4	0.018
Systolic blood pressure (mmHg)	128 ± 19	128 ± 21	0.93	132	125	+5.6	0.002
Diastolic blood pressure (mmHg)	76 ± 9	72 ± 10	<0.001	77.1	71.5	+7.8	<0.001
Antihypertensive use	79 (40.5)	428 (39.9)	0.87	53.0	36.4	+45.6	<0.001
Lipids							
Total cholesterol (mg/dl)	195 ± 38	197 ± 38	0.62	194	197	-1.5	0.41
LDL cholesterol (mg/dl)	116 ± 31	117 ± 32	0.53	115.2	117.6	-2.1	0.34
HDL cholesterol (mg/dl)	55 ± 19	52 ± 15	0.03	54.4	51.6	+5.4	0.020
Triglycerides (mg/dl)	126 ± 93	118 ± 72	0.25	101.2	103.8	-2.5	0.53
Lipid medication use	34 (17.4)	231 (21.5)	0.20	16.2	21.6	-25.0	0.10
Cigarette smoking							
Current	23 (11.8)	152 (14.4)	0.33	10.1	12.9	-21.7	0.30
Ever	115 (59.0)	591 (56.9)	0.60	59.2	57.4	+3.1	0.67
Body mass index (kg/m^2^)	28.4 ± 5.3	29.3 ± 5.7	0.034	28.8	29.3	-1.7	0.25
Waist circumference (cm)	95.7 ± 15.5	99.5 ± 14.7	0.002	96.9	99.3	-2.4	0.050
Any physical activity	144 (73.9))	832 (78.5	0.15	70.6	80.0	-11.8	0.010
Homocysteine (μmol/l)	9.45 ± 2.80	9.28 ± 3.23	0.45	9.49	8.88	+6.9	0.003
Serum inflammatory markers							
C-reactive protein (mg/l)	2.5 (1.1 to 7.2)	2.2 (1.0 to 4.7)	0.03	3.0	2.1	+44.0	<0.001
IL-6 (pg/ml)	3.9 (1.8 to 7.8)	1.3 (0.8 to 2.0)	<0.001	4.0	1.3	+208	<0.001
Fibrinogen (mg/dl)	335(278 to 416)	340 (294 to 390)	0.98	358	338	+5.9	0.001
sICAM-1 (ng/ml)	299(229 to 371)	261 (219 to 307)	<0.001	293	259	+13.1	<0.001
E-selectin (ng/ml)	49 (30 to 73)	50 (40 to 63)	0.46	50	49	+1.4	0.85
Composite risk factors							
Framingham 10-year risk^c ^(%)	7 ± 7	8 ± 7	0.04	9.35	8.25	+13.3	0.006
Reynolds Risk Score (women only) (%)	1.0 (0 to 2.0)	1.4 (0.2 to 2.7)	0.001	1.50	1.42	+5.6	0.60
Metabolic syndrome (Adult Treatment Panel III)	44 (22.6)	368 (34.5)	<0.001	24.4	34.0	-28.2	0.016

^a^Evaluation of Subclinical Cardiovascular Disease and Predictors of Events in Rheumatoid Arthritis (ESCAPE RA) study. Adjusted for age, gender and race. Characteristics expressed as the mean ± standard deviation, as *n *(%) or as the median (interquartile range). HDL, high-density lipoprotein; LDL, low-density lipoprotein; MESA. Multi-Ethnic Study of Atherosclerosis; RA. rheumatoid arthritis; sICAM-1, soluble intracellular adhesion molecule 1. ^b^Presence of hypertension defined as resting blood pressure >140/90 mmHg or use of antihypertensive medication. ^c^National Cholesterol Education Program criteria; excluding diabetics (fasting glucose ≥ 126 or use of diabetes medication).

Owing to imbalances in demographic characteristics between groups, mean CV risk factor levels by RA status were adjusted for age, gender, and race/ethnicity (Table 2). Thereafter, compared with controls, RA patients had significantly higher adjusted mean systolic and diastolic blood pressures, high-density lipoprotein-cholesterol, homocysteine, fibrinogen, and significantly lower fasting glucose, with percentage differences ranging from -8.2% (fasting glucose) to +7.8% (diastolic blood pressure). Adjusted mean CRP and IL-6 concentrations were 44% and 208% higher, respectively, in RA patients than controls. After adjustment, Framingham 10-year hard CV risk scores were significantly higher in the RA group by an average of 1.1 percentage points, while the mean Reynolds Risk Score (women only) did not significantly differ according to RA status.

### Association of RA status with the presence and extent of coronary arterial calcification

In crude comparisons, the prevalence of any CAC did not differ between the RA and MESA groups (55% vs. 56%, respectively). After sociodemographic and risk factor adjustment (Table 3, model 3), however, CAC prevalence was slightly higher in the RA group (prevalence ratio = 1.12; 0.05 <* P *< 0.10); this association was no longer present when IL-6 was entered into the model (Table 3, model 4). Stratification by gender revealed a significantly higher CAC prevalence in RA men after adjustment, but not in women, than in their controls – a heterogeneity that was statistically significant (*P *for interaction = 0.032). The heterogeneity was no longer significant after adjustment for IL-6 (Table 3, model 4).

**Table 3:** Crude and adjusted prevalence ratios for any coronary calcification (Agatston score > 0)^a^

RA vs. non-RA	Model 1	Model 2	Model 3^b^	Model 4
Total participants	0.98 (0.85 to 1.13)	1.08 (0.95 to 1.23)	1.12 (0.99 to 1.27)	1.00 (0.86 to 1.17)
Men only	1.15 (1.00 to 1.33)	1.16*(1.01 to 1.33)	1.19* (1.04 to 1.29)	1.14 (0.96 to 1.36)
Women only	0.87 (0.69 to 1.10)	1.06 (0.85 to 1.32)	1.09 (0.87 to 1.36)	0.89 (0.69 to 1.16)

^a^Prevalence ratios (95% confidence intervals) for rheumatoid arthritis (RA) participants and controls in the Evaluation of Subclinical Cardiovascular Disease and Predictors of Events in Rheumatoid Arthritis study. Prevalence ratios indicate the ratio of the prevalence of coronary artery calcification > 0 for RA participants compared with Multi-Ethnic Study of Atherosclerosis controls. Analyses include 1,222 subjects with complete data (RA, n = 193; control, n = 1,029). Model 1, crude model, no adjustment; model 2, adjusted for demographics (age, gender (where appropriate), race, and highest educational level attained); model 3, adjusted for model 2 covariates + cardiovascular risk factors (hypertension, high-density lipoprotein-cholesterol and low-density lipoprotein-cholesterol, diabetes, ever smoking, and use of lipid-lowering medication); model 4, adjusted for model 3 covariates + log IL-6. ^b^Additional covariates tested but noncontributory to the model: log CRP, log fibrinogen, log soluble intracellular adhesion molecule, triglycerides, exercise, body mass index, and waist circumference. *P value for the gender interaction significant at < 0.05.

**Figure 1:**
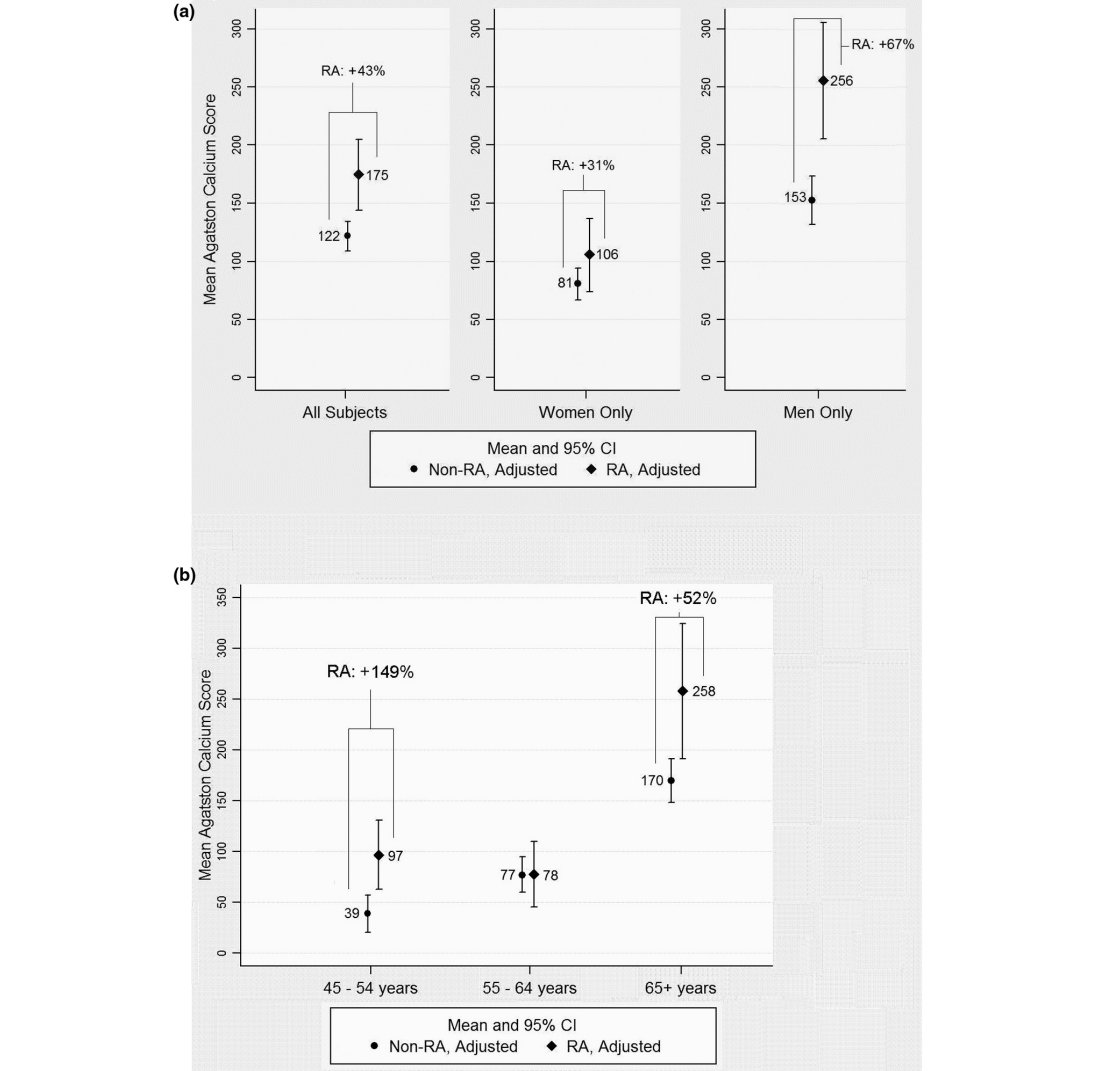
Adjusted mean Agatston calcium scores for participants with any coronary artery calcification (Agatston score > 0). Adjusted mean Agatston calcium scores for participants with any coronary artery calcification according to rheumatoid arthritis (RA) status and **(a) **gender and **(b) **age category. Analyses include 667 subjects with complete data. Analyses were adjusted for age, gender (where appropriate), race, hypertension, high-density lipoprotein-cholesterol and low-density lipoprotein-cholesterol, diabetes, ever smoking, and use of lipid-lowering medication. Means are enumerated and 95% confidence intervals (CIs) are indicated. *P *for gender interaction = 0.017; *P *< 0.05 for both age group interaction comparing the youngest or oldest age group with the middle age group.

In participants with prevalent CAC (Agatston score > 0), the CAC extent was significantly associated with increasing age, male gender, non-African American race, hypertension, diabetes, ever smoking, low-density lipoprotein-cholesterol, and RA status in multivariable analyses (data not shown). After adjustment for these demographic and CV risk factors, the mean adjusted CAC score (in those with an Agatston score > 0) was 53 units higher in the RA group overall compared with non-RA controls (Table 4, model 3; *P *= 0.002). Adding IL-6 into the model as a potential mediator attenuated this difference by an average of 10 units (18%) that, however, remained statistically significant (*P *= 0.028). Similar attenuation was observed when CRP was modeled as a potential mediator (data not shown). Differences in adjusted mean CAC scores were greater for men than for women with RA vis-à-vis their controls (Figure 1a; *P *for interaction = 0.017).

**Table 4:** Crude and adjusted associations of rheumatoid arthritis status with mean Agatston scores^a^

	Model 1	Model 2	Model 3	Model 4^b^
Characteristic	β (95% CI)	β (95% CI)	β (95% CI)	β (95% CI)
	
RA vs. MESA^c^	32.6* (2.04 to 64.1)	44.4* (12.3 to 76.4)	53.1* (19.8 to 86.4)	43.8* (4.9 to 82.8)

	Crude Mean (95% CI)	Adjusted Mean (95% CI)	Adjusted Mean (95% CI)	Adjusted Mean (95% CI)
	
RA CAC score^d^	145 (116 to 174)	162 (133 to 191)	175 (144 to 205)	166 (131 to 200)
MESA CAC score^d^	112 (100 to 125)	118 (105 to 130)	122 (109 to 134)	122 (109 to 135)

^a^Associations in subjects with prevalent coronary calcification in the Evaluation of Subclinical Cardiovascular Disease and Predictors of Events in Rheumatoid Arthritis study. Analyses include 667 subjects with Agatston coronary artery calcification (CAC) score > 0 and complete data. Model 1, crude model, no adjustment; model 2, adjusted for sociodemographics (age, gender, African American race, highest educational level attained); model 3, adjusted for model 2 covariates + cardiovascular risk factors (hypertension, diabetes, smoking, high-density lipoprotein-cholesterol and low-density lipoprotein-cholesterol, and lipid lowering medication use); model 4, adjusted for model 3 covariates + log IL-6. ^b^Additional covariates tested but noncontributory to the model: log C-reactive protein, log fibrinogen, log soluble intracellular adhesion molecule, log homocysteine, exercise, body mass index, and waist circumference. ^c ^β coefficients (95% confidence intervals) representing the difference in mean Agatston CAC score for rheumatoid arthritis (RA) participants vs. Multi-Ethnic Study of Atherosclerosis (MESA) controls from robust regression models. * β coefficient significant at *P *< 0.05.^d^Values represent crude (model 1) and adjusted (models 2 to 4) mean (95% confidence interval) coronary artery calcification scores per group (RA or MESA) under the robust regression model above.

Differences in mean Agatston scores by RA status were also heterogeneous by age category (Figure 1b). Significantly higher adjusted mean Agatston scores were observed for RA patients compared with controls in the youngest (45 to 54 years) and oldest (65+ years) age groups, but not in the middle age group (55 to 64 years) (*P *value for age group interaction comparing the youngest or oldest age group with the middle age group < 0.05 for both). The greatest percentage difference in mean adjusted Agatston scores between RA patients and controls was observed in the youngest age group, in which Agatston scores were 2.5-fold higher for RA patients.

### Association of RA severity with the presence and extent of coronary arterial calcification

**Figure 2:**
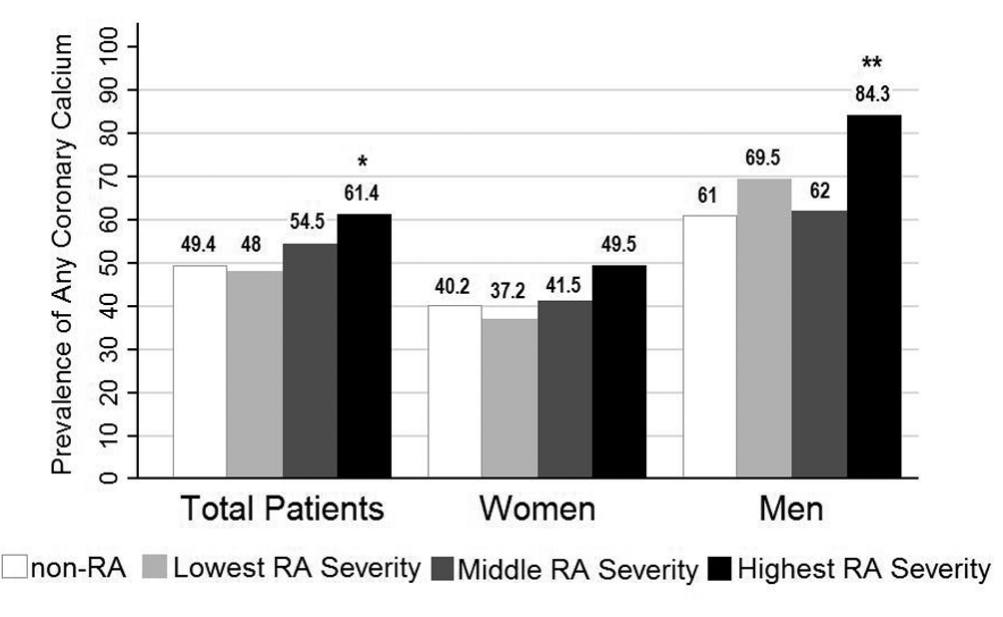
Adjusted associations of rheumatoid arthritis severity with prevalence of coronary artery calcification (Agatston score > 0). Tertiles of rheumatoid arthritis (RA) severity according to gender are gender-specific. Prevalence of any coronary calcium is given as a percentage of the total. Adjusted comparisons include age, gender (where appropriate), race, highest education level attained, hypertension, high-density lipoprotein-cholesterol and low-density lipoprotein-cholesterol, diabetes, ever smoking, and use of lipid-lowering medication. Analyses include 1,222 subjects with complete data (RA, n = 193; control, n = 1,029). *P < 0.05 compared with the non-RA group. **P < 0.05 compared with both the non-RA group and the lowest tertile of RA severity.

The CAC prevalence increased as the propensity scores for RA disease activity and severity increased (Figure 2). After adjusting for sociodemographics and CV risk factors, the prevalence of any CAC for RA patients in the lowest tertile of RA severity did not significantly differ from that of MESA controls (48% vs. 49.4%, respectively); however, the adjusted CAC prevalence was 61.4% in RA patients in the highest tertile of RA severity, a difference that was statistically significant compared with MESA controls (*P *= 0.021). Notably, the trend of increasing prevalence of any CAC with increasing RA severity was observed for both men and women with RA.

**Figure 3:**
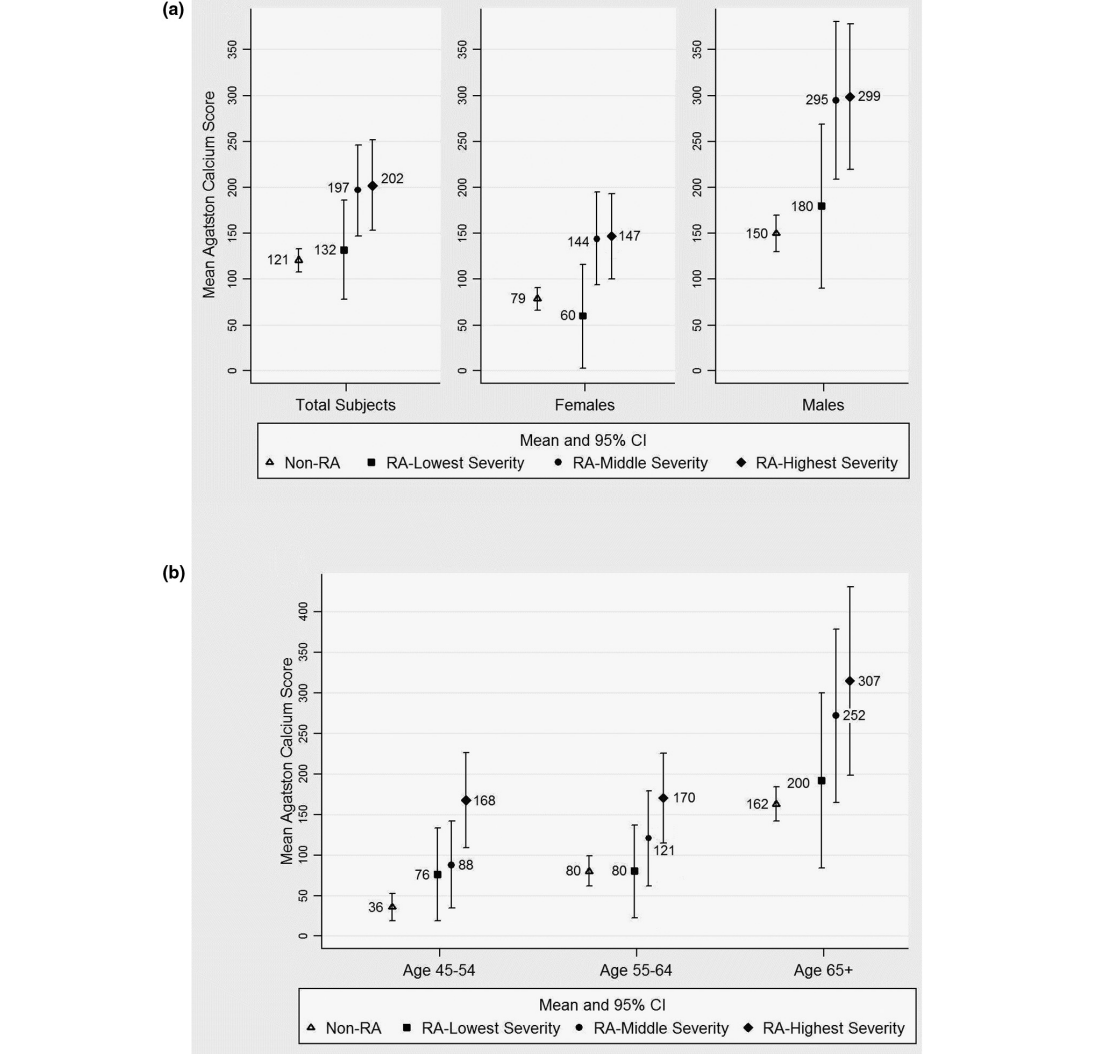
Adjusted mean Agatston scores for participants with coronary artery calcification according to rheumatoid arthritis severity. Adjusted mean Agatston calcium scores for participants with any coronary artery calcification (CAC) (Agatston score > 0) according to tertiles of rheumatoid arthritis (RA) severity, by **(a) **gender and **(b) **age category. Analyses include 667 subjects with complete data. Tertiles of propensity for RA disease activity and severity according to age category are gender-specific. Analyses were adjusted for age, race/ethnicity, hypertension, high-density lipoprotein-cholesterol and low-density lipoprotein-cholesterol, diabetes, ever smoking, and use of lipid-lowering medication. Means are enumerated and 95% confidence intervals (CIs) are indicated. *P *values for the linear trend of increasing RA severity with CAC are < 0.05 for the total group and in all subgroup analyses.

Increasing RA severity was associated with higher mean CAC scores in participants with prevalent CAC (Agatston score > 0), such that the difference in adjusted mean CAC scores between the lowest RA severity tertile and controls was small and nonsignificant, but the difference was large and significant between the middle–upper severity tertiles and MESA controls (Figure 3a). For example, RA patients in the highest tertile of RA severity had an adjusted Agatston score of 202 versus only 121 in controls (*P *= 0.006). This pattern was seen in both genders (Figure 3a) and across all age groups (Figure 3b). No statistically significant interactions were seen between RA severity and gender or age.

## Discussion

In the present study – the largest study to date investigating CAC in RA – we observed a higher prevalence and extent of CAC in RA patients compared with a geographically compatible population of non-RA controls after CV risk and sociodemographic adjustments. These associations were attenuated to varying degrees in models including IL-6, a marker of system inflammation – suggesting that IL-6 is a potential mediating variable. Increasing overall RA severity was also associated with a higher prevalence and extent of CAC, further suggesting that the RA disease process contributes to atherogenesis independently of traditional CV risk factors. We also observed age and gender heterogeneities with potential implications for RA patients. In particular, differences in CAC between men with RA and male controls were greater than those for women. In addition, the largest percentage difference in CAC between RA patients and their non-RA counterparts was observed in the youngest age category.

Two previous studies explored CAC in RA [5,6]. Chung and colleagues investigated whether RA duration was associated with a higher CAC prevalence by enrolling only patients with either early RA (RA duration <5 years) or established RA (RA duration > 10 years), and compared these groups with a small group of non-RA controls [5]. A greater prevalence of CAC was observed in the established RA group compared with both the control and early RA groups after adjusting for demographic and current CV risk factors. Their study did not, however, include a sufficient sample of men to analyze this group separately. In the study by Kao and colleagues, an association between the RA duration and the extent of CAC was observed, independently of demographic and CV risk factors [6]. Their study did not incorporate a non-RA comparison group, however, and only women were enrolled. The present report differs from these prior studies in several important ways. The current study's larger size and comparison with MESA allowed subgroup comparisons by gender and age category that revealed potentially important heterogeneities. In addition, our exclusion of prior CV events allowed a focus on subclinical disease, thus limiting potential skewing of CAC in groups with prior CV events and procedures.

The mechanism underlying the observed increase in CAC in RA is likely complex and multifactorial. In multiple population-based studies [31-33], individuals with the highest concentrations of circulating inflammatory markers were at greatest risk for CV events and mortality, and tended to demonstrate an increased burden of subclinical atherosclerosis, including CT-measured CAC [34]. A direct effect of inflammatory cytokines on the vasculature in promoting atherogenesis and destabilizing coronary plaques has been proposed as a potential mechanism [13]. RA patients have considerably higher circulating levels of inflammatory cytokines than non-RA controls, and adjustment for IL-6 in the statistical models partially attenuated the observed association between RA status and CAC, suggesting that systemic inflammation accounted for at least part of the association. We did not, however, see the same magnitude of attenuation upon adjustment for serum CRP concentration, which has been associated with CV events and subclinical atherosclerosis [31,34]. While this apparent disconnection might suggest pathogenic specificity of individual inflammatory cytokines, these speculations are limited by the cross-sectional nature of the analysis, and the discrepancy may be the result of random variability. Importantly, a single measurement of inflammatory cytokines is not representative of the total inflammatory burden of RA patients. While follow-up will help to assess the associations of cumulative inflammation with progression of CAC in RA patients, the cross-sectional association of RA propensity scores (a clinical reflection of current and past burden of disease) with CAC nonetheless supports an association between inflammation and CAC. On the other hand, finding that the association of RA status and CAC is only partially attenuated by IL-6 suggests the presence of additional RA-related mediators. These might include a phenotypically unusual T-cell clone (CD4^+^CD28^-^) [35], shared risk factors (such as genetic predisposition), RA treatments (such as glucocorticoids and nonsteroidal anti-inflammatory drugs), and the debilitating effect of joint pain and stiffness on physical activity and fitness.

In most studies of CVD in RA, differences in the prevalence of traditional CV risk factors compared with controls have not been detected [11,12]. We detected small but significant differences in blood pressure in RA patients compared with controls that could affect CV risk unfavorably. We also detected higher mean high-density lipoprotein-cholesterol, lower mean fasting glucose and diagnosed diabetes, and lower prevalence of the metabolic syndrome in the RA groups, however – all of which could decrease CV risk. Although heterogeneity in the associations with traditional CV risk factors, other than age and gender, on CAC by RA status was not explored, our findings suggest that conventional means of risk-stratifying RA patients probably underestimate their risk. In particular, the largest difference in CAC, by a several-fold increase, between RA patients and controls was observed in the youngest age category, an age group in which CAC is typically low in both men and women [16]. This same heterogeneity by age has also been observed for carotid plaque in a study primarily of women with RA [4], in which the largest percentage difference in the prevalence of carotid plaque was observed in the RA patients younger than age 50 years.

There are some notable limitations to our study. As MESA is a population-based study without exclusion of patients with rheumatic disease, it is possible that RA patients could have been included in the non-RA control group. As such potential misclassification would tend to lessen the observed differences between the RA and control groups, the true effect of complete accuracy in the classification of exposure status would actually strengthen the association of RA status with CAC. To reduce misclassification by RA status, we excluded patients from the control group who reported using medications commonly used to treat active RA. While this method of identification of RA is more reliable than patient self-report of the diagnosis [36] and is commonly used in epidemiological studies for the diagnosis of RA, there are limitations – RA patients not taking DMARDs would be included, and those with other diseases in which these medications are used, such as Crohn's disease or psoriasis, would be excluded. Another limitation is that calcification of coronary plaque may not be equally representative of the same atherosclerotic burden in RA patients as controls, since noncalcified soft plaque is not detected by Agatston scoring [37]. This limitation is supported by a recent autopsy study in which RA patients demonstrated scattered vulnerable plaques that were highly inflammatory on histologic examination despite having less extensive atherosclerotic burden overall compared with controls [7]. Finally, differences in referral patterns into the study (community based for the MESA vs. clinic based for the ESCAPE RA study) could have introduced selection bias or confounding on factors not related to exposure status. However, as the two cohorts were geographically compatible, as all of the ESCAPE RA patients were community dwelling, and as many ESCAPE RA patients were recruited from community rheumatologists, it is likely that any bias related to selection would be limited. Another potential problem is that the analyses were cross-sectional, and thus temporality cannot be established.

## Conclusions

In summary, we observed a greater prevalence and extent of CAC in RA patients compared with controls even after adjusting for key confounders. In addition, associations were linked to overall RA severity and were of greater magnitude for men and younger patients. Interventional trials targeted at tight control of RA and at aggressive monitoring and management of CV risk factors are needed to identify the most effective means of reducing CV risk in this high-risk population.

## Abbreviations

CAC: coronary artery calcification; CRP: C-reactive protein; CT: computed tomography; CV: cardiovascular; CVD: cardiovascular disease; DMARD: disease-modifying antirheumatic drug; ELISA: enzyme-linked immunosorbent assay; ESCAPE RA: Evaluation of Subclinical Cardiovascular disease And Predictors of Events in Rheumatoid Arthritis; IL: interleukin; MESA: Multi-Ethnic Study of Atherosclerosis; RA: rheumatoid arthritis.

## Competing interests

The authors declare that they have no competing interests.

## Authors' contributions

JMB, JTG, MS, WP, MP, RSB, ACG and RD were involved in the study design. JMB, JTG, RD, WWS and GL were involved in the acquisition of data. JMB, JTG, RAK, MS, WP, RSB, MP and ACG were involved in the data interpretation and analysis. All authors were involved in manuscript preparation.
